# Bioequivalence of two formulations of montelukast sodium 4 mg oral granules in healthy adults

**DOI:** 10.1186/2045-7022-4-29

**Published:** 2014-09-18

**Authors:** Constanze Fey, Ursula Thyroff-Friesinger, Spencer Jones

**Affiliations:** 1 Hexal AG, Holzkirchen, Germany; 2 Sandoz International GmbH, Holzkirchen, Germany

**Keywords:** Montelukast, Bioequivalence, Asthma, 4 mg oral granules, Sandoz, Singulair^®^ mini, Rhinitis

## Abstract

Montelukast is an effective and well-tolerated treatment for the prophylaxis and chronic treatment of asthma, acute prevention of exercise-induced bronchoconstriction and symptomatic relief of seasonal allergic rhinitis and perennial allergic rhinitis. The aim of the study was to compare bioavailability, and characterise the pharmacokinetic profile and safety of Sandoz generic montelukast 4 mg oral granules relative to Singulair^®^ mini (Merck, Sharp & Dohme). An open-label, randomised, single-dose, two-treatment, two-period, two-sequence, two-way crossover bioequivalence study was conducted in healthy male volunteers aged 18–55 years, under fasting conditions. The duration of the clinical part of the trial was ≈ 11 days. Montelukast levels in plasma were quantified using a validated liquid chromatography tandem mass spectrometry method, and pharmacokinetic parameters calculated from the drug concentration–time profile using a non-compartmental model. A total of 40 subjects completed both study periods. The ratio test/reference of geometric least squares means was calculated for both formulations of montelukast for the In-transformed pharmacokinetic parameters; the 90% confidence intervals (CIs) were within the pre-defined limits of 80.00–125.00%: 92.2% (90% CI: 87.42–97.30%) for C_max_, 98.1% (90% CI: 94.49–101.81%) for AUC_0–t_ and 97.6% (90% CI: 94.14–101.27%) for AUC_0–∞_. Two study subjects each reported one mild adverse event: dyspepsia (possibly related to study medication) and throat pain (not considered related to study medication). Sandoz montelukast 4 mg oral granules are bioequivalent to Singulair^®^ 4 mg mini oral granules, with a similar safety profile. This suggests that these two preparations can be considered interchangeable in clinical practice.

## Findings

The leukotriene receptor antagonist montelukast, administered once daily as either 10 mg or 5 mg tablets, 5 mg or 4 mg chewable tablets, or oral granules at a dose of 4 mg, is an effective and well-tolerated treatment for the prophylaxis and chronic treatment of mild asthma in patients aged ≥2 years, the acute prevention of exercise-induced asthma in patients aged ≥6 years, and the symptomatic relief of seasonal allergic rhinitis in individuals aged ≥2 years, and perennial allergic rhinitis on those aged ≥6 months [1,2]. In patients aged 6–24 months, the 4 mg oral granules preparation has been demonstrated to provide systemic exposure similar to that of the 10 mg film-coated tablet in adults [3].

Sandoz developed a montelukast 4 mg oral granules formulation containing the same active ingredient and excipients as Singulair^®^ mini (4 mg oral granules) [1,2], which was approved by the US Food and Drug Administration for the treatment of asthma and allergic rhinitis in August 2012 [2,4]. Singulair^®^ mini is primarily indicated in the treatment of asthma as add-on therapy in those 6 months to 5 year old patients with mild to moderate persistent asthma who are inadequately controlled on inhaled corticosteroids and in whom “as-needed” short acting β-agonists provide inadequate clinical control of asthma [2].

The aim of this study was to compare the bioavailability and characterise the pharmacokinetic profile of the Sandoz montelukast formulation relative to Singulair^®^ mini and to assess the bioequivalence of the two formulations. The secondary objective was to investigate the safety of the two formulations, on the basis of clinical and laboratory examinations and documentation of adverse events.

## Methods

This study was completed in accordance with Good Clinical Practice, Good Laboratory Practice, the Declaration of Helsinki and the European Medicines Agency (EMA) bioequivalence guidance. The Independent Ethics Committee – Aditya (IEC-A) provided approval on 5 June 2008.

This bioequivalence study was conducted as a monocentric open-label, randomised, single-dose, two-treatment, two-period, two-sequence, two-way crossover study of two formulations of montelukast 4 mg oral granules between 9 and 20 June 2008. Screening was scheduled within 21 days prior to dosing day and all study subjects fasted for at least 10 hours prior to drug administration. Subjects were randomised to either Singulair^®^ mini 4 mg oral granules (treatment A) or Sandoz montelukast 4 mg oral granules (treatment B) (according to a randomisation schedule of sequence AB or BA). The 4 mg dose of Sandoz montelukast was used in this study instead of the 10 mg film-coated tablet to allow the test product to be compared with the corresponding dosage and formulation of the reference product.

The duration of the trial was approximately 11 days, lasting from 11 hours prior to the administration of the first dose (period 1) until 36 hours after the administration of the second dose (period 2) with a washout phase of 9 days between the two periods. Venous blood samples were withdrawn within 60 minutes prior to dosing and at 0.25, 0.5, 0.75, 1, 1.25, 1.5, 1.75, 2, 2.25, 2.5, 2.75, 3, 3.25, 3.5, 3.75, 4, 4.5, 5, 5.5, 6, 8, 10, 12, 16, 24 and 36 hours after each dose. No blinding was implemented during drug administration, but the analyst was held blind. The study was conducted in healthy, male volunteers, aged 18–55 years. Additional inclusion criteria included a body mass index (BMI) of 19–27 kg/m^2^, and no significant diseases or clinically significant abnormal findings during screening. Exclusion criteria included, amongst others, any known hypersensitivity or idiosyncratic reaction to the study drug, history of asthma, the presence of clinically significant abnormal laboratory values during screening, recent history of alcoholism (<2 years) or of moderate (180 ml/day) alcohol use or consumption of alcohol within 48 hours prior to the first dose of study medication, and participation in another clinical trial within 90 days prior to the first dose of study medication. Additionally, any medication use 14 days prior to dosing was checked and subjects were instructed not to take any medicine during the study. Owing to methodological and ethical difficulties with pharmacokinetic studies in the paediatric population, EMA guidelines state that pharmacokinetic studies of drugs intended for use in a paediatric population can be carried out on healthy adults [5]. Results are typically extrapolated to the population for which the reference product is approved [5]. Separate bioequivalence studies were performed for Sandoz montelukast film coated tablets versus the Singulair 10 mg film coated tablets [Sandoz, Data on File].

A total of 42 subjects were enrolled, together with two additional subjects to account for the possibility of any discontinuations prior to the administration of the first dose. Based on the half-life of the drug (2.7–5.5 hours in healthy young adults), a washout period of 7–14 days was considered appropriate. The pharmacokinetic profile of the Sandoz formulation (test) was characterised relative to that of Singulair^®^ mini (reference) and the bioequivalence of the two products was assessed. The crossover study design meant that each subject acted as his own control, negating the need for a control group. Each subject received a single dose (one sachet containing 4 mg oral granules) of Sandoz montelukast (test) and Singulair^®^ mini (Merck Sharp & Dohme BV Nederland; reference). The relevant products were administered to each subject on 10 June 2008 (period 1), and on 19 June 2008 (period 2), according to the randomisation schedule. Whole blood samples were collected prior to dosing, and over a period of 36 hours following each dose. Plasma levels of montelukast sodium were quantified using a validated liquid chromatography tandem mass spectrometry method and the following pharmacokinetic parameters calculated from the drug concentration–time profile by non-compartmental modelling: T_max_, C_max_, AUC_0–t_, AUC_0–∞_, λ_z_, t_½_, and residual area. The following evaluations were made to assess the safety and tolerability of montelukast: clinical examination together with recording of vital signs, including measurement of oral body temperature; sitting blood pressure and radial pulse; X-ray; electrocardiogram; clinical laboratory parameters (biochemistry, haematology, urinalysis) and adverse event monitoring. Based on in-house estimates, the maximum intra-subject variability observed for the primary pharmacokinetic parameter for montelukast was approximately 26%. Assuming an intra-subject coefficient of variation of approximately 26% for both AUC and C_max_, and with an expected ratio of 0.95–1.05 for AUC and C_max_, the study should have a power of at least 80% to show bioequivalence of the two formulations with 36 subjects. However, to allow for dropouts and withdrawals, 42 volunteers were recruited for the study.

Analysis of variance, two one-sided tests for bioequivalence, power and ratio analyses for un-transformed and In-transformed pharmacokinetic parameters Cmax, AUC0–t and AUC0–∞ were computed for montelukast. The 90% parametric confidence intervals were calculated for the un-transformed and In-transformed pharmacokinetic parameters of the montelukast data. Bioequivalence was concluded if the 90% confidence intervals of the ratio test/reference of geometric least squares means fell within the acceptance range of 80.00–125.00% for In-transformed pharmacokinetic parameters C_max_, AUC_0-t_ and AUC_0-∞_ for montelukast.

## Results

**Figure 1:**
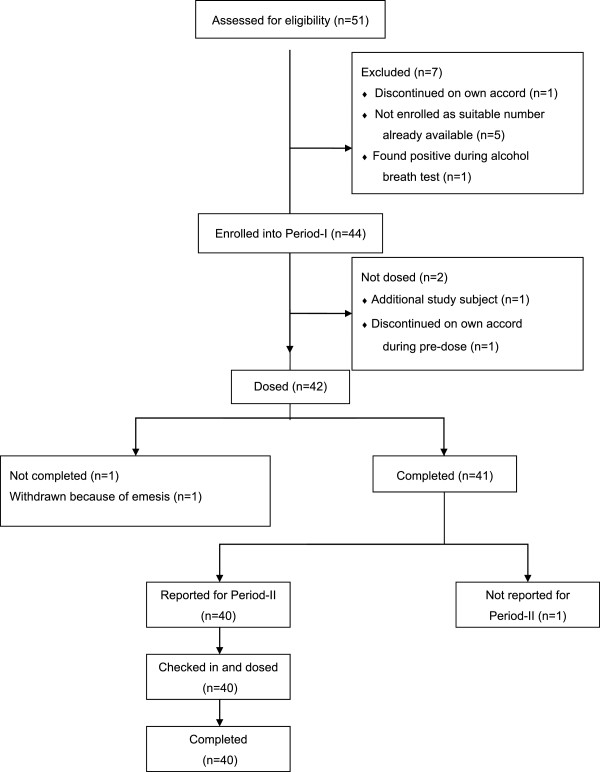
Study subject disposition.

**Figure 2:**
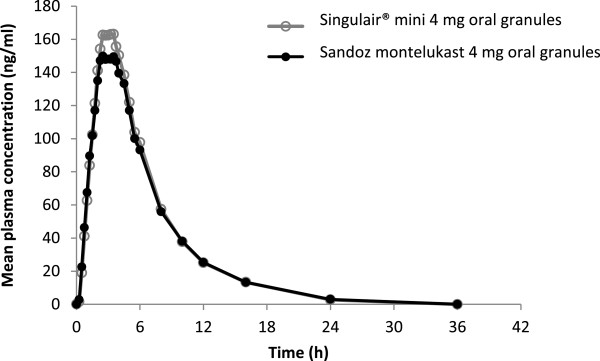
Montelukast plasma concentration versus time profile following administration of Singular^®^ and Sandoz montelukast 4 mg oral granules.

As per the study protocol, 42 subjects were dosed in the first period, however, 40 subjects were dosed in the second period (Figure 1); one was withdrawn on the grounds of emesis after the first dose, while the second subject discontinued of his own accord on the check-in day for the second dose. Plasma samples of the 40 successful completer subjects were analysed. Treatment compliance was confirmed by examination of the oral cavity immediately after dosing. Enrolled subjects had a mean age of 28.3 years and a mean BMI of 21.42 kg/m^2^ (Table 1). Pharmacokinetic parameters were derived for each individual from the concentration versus time profiles of montelukast in plasma. The descriptive statistics of pharmacokinetic parameters for test and reference for the 40 study subjects are summarised in Table 2; the ratio test/reference of the geometric least squares means for C_max,_ AUC_0–t_ and AUC_0-_∞. along with their 90% CIs are shown in Table 3. The mean plasma concentrations of montelukast over time post-administration of the two formulations are shown in Figure 2. The ratio for key pharmacokinetic parameters for both formulations of montelukast was 92.2% for C_max_ (90% CI: 87.42–97.30%), 98.1% for AUC_0–t_ (90% CI: 94.49–101.81%) and 97.6% for AUC_0-∞_ (90% CI: 94.14–101.27%). Two mild adverse events in two separate subjects were reported during the study; one occurred during Period 1 and the second during the subsequent washout phase. Both events occurred in subjects receiving Sandoz montelukast and were fully resolved. One was dyspepsia and considered possibly drug related; the other was throat pain and considered unlikely to be drug related. There were no serious or significant adverse events or deaths, with both formulations being well tolerated when administered as a single dose.

**Table 1:** Baseline study subject demographics

	**Mean ± SD**	
**Parameter (Units)**	**n = 42 (Dosed subjects)**	**n = 40 (Study completers)**
**Age (years)**	28.3 ± 7.02	28.5 ± 6.85
**Weight (kg)**	60.35 ± 7.17	60. 20 ± 6.92
**Height (cm)**	167.80 ± 6.30	167.33 ± 6.12
**BMI (kg/m**^**2**^**)**	21.42 ± 2.14	21.48 ± 2.05

BMI: body mass index.

SD: standard deviation.

**Table 2:** Descriptive statistics of formulation means for montelukast (n = 40)

**Parameters (Units)**	**Mean ± SD (un-transformed data)**	
	**Sandoz montelukast 4 mg oral granules**	**Singulair^®^ mini 4 mg oral granules**
T_max_ (h)*	2.75	2.75
C_max_ (ng/ml)	178.04 ± 41.58	196.04 ± 53.25
AUC_0–t_ (ng.h/ml)	1083.00 ± 293.88	1112.84 ± 309.13
AUC_0-_∞ (ng.h/ml)	1134.70 ± 305.17	1170.45 ± 321.90
λ_z_ (1/h)	0.17 ± 0.038	0.18 ± 0.042
t_½_ (h)	4.36 ± 1.00	4.04 ± 1.06
Residual area	4.71 ± 1.86	5.13 ± 2.32
(AUC_% Extrap_Obs (%))		

*median value.

T_max_: time of the maximum measured plasma concentration.

C_max_: maximum measured plasma concentration.

AUC_0–t_: the area under the plasma concentration versus time curve from time zero to the last measurable concentration.

AUC_0–∞_: the area under the plasma concentration versus time curve from time zero to infinity.

AUC_% Extrap_Obs (%): % of the AUC that has been derived after extrapolation.

λ_z_: first order rate constant associated with the terminal (log-linear) portion of the curve.

t_½_: the elimination or terminal half-life.

**Table 3:** Geometric least squares mean, ratio and 90% CI for montelukast (n = 40)

**Parameters (Units)**	**(In-transformed) Geometric least squares mean**			**90% CI (parametric)**
	**Sandoz montelukast 4 mg oral granules**	**Singulair^®^mini 4 mg oral granules**	**Ratio (%)**	
C_max_ (ng/ml)	171.76	186.23	92.2	87.42–97.30%
AUC_0–t_ (ng.h/ml)	1041.90	1062.31	98.1	94.49–101.81%
AUC_0-_∞ (ng.h/ml)	1093.73	1120.19	97.6	94.14–101.27%

C_max_: maximum measured plasma concentration.

AUC_0–t_: the area under the plasma concentration versus time curve from time zero to the last measurable concentration.

AUC_0-_∞: the area under the plasma concentration versus time curve from time zero to infinity.

## Discussion

The findings of this study confirmed 90% confidence intervals of 87.42–97.30%, 94.49–101.81% and 94.14–101.27% for C_max_, AUC_0–t_ and AUC_0-_∞, respectively, all of which are within the predefined bioequivalence acceptance limits of 80.00–125.00%, according to the relevant guideline of the EMA Committee for Medicinal Products for Human Use [6]. The demonstration of bioequivalence between these two preparations of montelukast, may lead to an increased range of available preparations of oral granules of montelukast. Owing to the lower investment costs associated with generic products such as the Sandoz montelukast, introduction of this generic monteulast product could have cost-effectiveness and budgetary implications [7].

The limitations of the current study are the relatively small sample size and administration of a single-dose in healthy male volunteers. Several clinical studies have demonstrated the efficacy of Singulair^®^ mini in the treatment of asthma in both adults and children [8-12], and for the symptomatic relief of allergic rhinitis [13-15]. Given the bioequivalence demonstrated for the Sandoz montelukast 4 mg oral granules, this formulation is expected to be equally efficacious and well-tolerated.

The current study has clearly demonstrated that Sandoz montelukast 4 mg oral granules are bioequivalent to Singulair^®^ mini 4 mg oral granules in terms of the rate and extent of absorption of each formulation. Sandoz montelukast 4 mg oral granules offer an efficacious and well-tolerated treatment option for individuals with asthma or seasonal/perennial allergic rhinitis.

## Competing interests

CF and UTF are employees of Hexal AG, an affiliate of Sandoz International GmbH, both Novartis companies. SJ is an employee of Sandoz International GmbH.

## Authors’ contributions

All authors contributed towards analysis and interpretation of the data, and drafted or critically revised the manuscript for important intellectual content. All authors approved the final version to be published.
